# Outdoor Therapy: An Interpretative Phenomenological Analysis Examining the Lived-Experience, Embodied, and Therapeutic Process through Interpersonal Process Recall

**DOI:** 10.3390/sports7080182

**Published:** 2019-07-25

**Authors:** Heidi Schwenk

**Affiliations:** School of Adventure Studies, West Highland College, University of the Highlands and Islands, Scotland PH33 6FF, UK; heidi.schwenk.whc@uhi.ac.uk

**Keywords:** outdoor therapy, phenomenology, therapeutic process, embodiment, lived-experience

## Abstract

This research explores an innovative methodology for understanding the process and practice of UK-based outdoor therapists. Recent studies address the need to expand circles of knowledge, and capture the lived-experience of outdoor practitioners to examine the ‘altered’ therapeutic process and frame. Interpersonal process recall (IPR) methodology offers a nuanced and contextualised lived-experience of outdoor therapists. IPR includes three phases: (1) initial-interview; (2) post-session-reflective-recording; and (3) an IPR-interview to replay and explore the participants’ recorded reflections of the outdoor therapy session. The sample included three UK-based outdoor therapists. Interpretative phenomenological analysis was used to qualitatively analyze the data. The study presents the theme of ‘transitional landscapes—transitional thinking’, which explores the embodied experience, the parallel process between the client and therapist, and watching for drift. The findings provide insight for training and supervision and generates constructive dialogue amongst outdoor therapists. The research supports IPR as a methodology offering participant and researcher experiential and reflective positions. Parallels are drawn in relation to existing research, literature, and contemporary professional issues surrounding outdoor therapy as a mental health treatment.

## 1. Outdoor Therapy

The search for common meaning in outdoor therapy has proven to be challenging [1]. Discussions across the world have sought, and continue to seek, a mutual definition for outdoor therapy [2,3]. Whilst acknowledging the multiple definitions and forms of practice in the UK, this study attempts to narrow the focus to outdoor therapy, which includes a range of constructs relating to natural and wild places. This encompasses a variety of outdoor activities and therapeutic modalities to integrate a three-way relationship between the client, practitioner, and the environment. Johnson [4] (p. 72) suggests a definition of outdoor therapy in the UK based on a multidisciplinary forum held in 2006 by the University of Central Lancashire, which invited the British Association for Counselling and Psychotherapy (BACP), the United Kingdom Council for Psychotherapy (UKCP), and the Institute for Outdoor Learning (IOL). Johnson [4] (p. 72) states that outdoor therapy:

“(1) Uses a process of supported self-discovery to promote wellbeing and change.

(2) Has some experience that takes place out-of-doors (recognition of interconnection to the environment and other themes).

(3) Recognizes the outdoor place is an active component in the therapeutic process and that the process involves other components such as place, experience and reflection.

(4) Understands that reflection (not reviewing) for the therapist and client is an integral part of the process and that these reflective processes include what is happening for both the therapist/practitioner and the client and their relationship to the outdoor place.”

### 1.1. The Role of the Therapist

Outdoor therapy brings together the intrinsic benefit received from outdoor environments with an intentional therapeutic approach. Nature-based interventions are emerging, offering health benefits and well-being outcomes, at the same time as cost effective alternative psychological therapies are being explored. “There are now numerous local and national organisations offering a range of nature-based interventions as specifically designed and structured health or social care treatment interventions” [5] (p. vi). Despite the growing demand and provision, there is limited literature surrounding the process of working with nature in therapy [6], the therapeutic frame, and practice issues [3]. The literature lacks an understanding of the role of the outdoors, looking at nature as a place to be utilized rather than central to the therapeutic process [7].

Outdoor therapy alters the therapeutic alliance (professional relationship) between the client and the therapist. In outdoor therapy, the outdoors is described as an intimate other within the counselling relationship; a third entity which provides a dyadic encounter, embodied, multi-sensory, here-and-now experience that differs to traditional therapy within a consultation room [8]. As such, outdoor therapy shifts the professional role of the therapist, allowing them to experience a new, revitalized definition of themselves with clients, which is more collaborative, dynamic, and emergent. McKinney [9] suggests the neutral and shared outdoor space and use of physical activities encourages a collaborative framework of practice. Therapists become responsive to the client’s needs, promoting empowerment, equality, and client-centred practice [1]. The therapeutic space “collaboratively emerges, is constantly negotiated and is unique with each client” [1] (p. 10). McKinney [9] suggests that working in this way can speed up the pace at which the client and therapist begin to engage with presenting issues. However, there remains a lack of contextualized accounts explaining this process and its impact on intimacy and the therapeutic relationship. Whilst previous studies have contributed to understanding the altered client–therapist dynamic [3,7,9,10], there remains scope to understand the lived-experience of these encounters in greater depth and gain nuanced accounts from practitioners [6]. Similarly, Revell and McLeod [1] encourage researchers to investigate an accurate perception of both positive and negative accounts from practice to further discussion around best-practice.

An understanding of outdoor therapy approaches depends on in-depth analysis of present approaches and processes in action. The therapeutic process has multiple meanings; the process of change, the ingredients that contribute to outcomes, the abstraction and conceptualization of experience, and applied skills in context [11]. Bragg and Atkins [5] advocate that the knowledge of process is vitally important as the outdoor therapy field strives to become recognized as a viable therapeutic treatment and alternative to mainstream therapies. Understanding the process is essential for gaining funding [11], and for offering clear training, guidance, supervision, and discussion for practitioners using the outdoors [1]. Crucially, it is important to offer transparency and clarity to clients about the outdoor therapy approach [11].

### 1.2. The Outdoor Environment: Figure or Ground?

Within the field of outdoor therapy, the outdoor environment has a wide range of applications. There is some debate on whether outdoor therapy is a modality of its own or an integrative approach, combining theories and practices from multiple modalities [1,9], and whether outdoor therapy is used as a task, goal, or method within the therapeutic process. As an idiosyncratic field, the application of outdoor therapy seems to depend on the practitioner’s personal background, training, and discipline. Berger and McLeod [12] advise therapists consider the role of nature; whether they are working in the foreground; adaptively as an active part of the process, the backdrop; space to journey within the therapeutic relationship; or using nature as a container, witness, or mediator. They suggest it is possible to shift between these uses of the outdoors as appropriate to the client’s needs [12], and understand the evolving interplay between the outdoor environment, therapist, and client. Within gestalt psychotherapy, the therapist responds to both the figure (background and context of the client) and ground (presenting issues that emerge) to focus the work whilst being mindful of the whole [13,14]. With outdoor therapies, the figure and ground take on new meaning and become experiential, metaphorical, and industrious [15]. As such, a “tripartite therapeutic partnership” is formed between the client, nature, and therapist whereby each can be affected by the others [6] (p. 66). 

Outdoor therapists can help clients access an embodied experience, where sensual and cognitive epistemologies can be explored [16]. “To ‘walk-and-talk’ is to harness an interplay between physical movement and therapeutic conversation in the outdoors that results in an integration of space, place and embodied experiencing” [17] (p. 10). The altered physicality of working outdoors offers nonverbal synchronicity between the client and therapist [1], contributes to a greater abstract conceptualisation [18,19], increases thought processes which can loosen stuck thinking and forge new connections between different concepts [20], and can exaggerate passions, mystical experiences, and sensory appreciation [21]. From a cognitive perspective, Gibbs et al. [22] suggest embodied experiences can alter the clients use of language and allow them to articulate affective and metaphoric connections which were previously inaccessible. Physical movement can facilitate a transition from the internal stuck place as it encourages creative freedom [1]. Central to the therapeutic process are the aspects of change within the modality and the therapist’s ability to use these elements in-the-moment. These aspects can range dramatically, such as adventure therapy, which emphasizes adventurous activities as the stimulus for change, compared to wilderness therapy, which favors solo time for reflection [1]. Conversely, some outdoor therapies, such as nature-based therapy, ecotherapy, and nature therapy, consider the natural environment as fundamental within the therapeutic process [1]. In most cases, the therapist’s modality reflects the benefits they see in the use of landscapes [23]. Whilst a pluralistic view might consider, “clients benefit from different things at different stages in their therapy” [17], it is noted the word ‘things’ in the field of outdoor therapy has a broad interpretation. 

Taking therapy outside can introduce an unpredictability and uncertainty within the therapeutic experience and is likely to alter the therapeutic frame [24]. Nature can be used as a container and safe place for the process to unfold [3] and can help the client find connection with their body, soul, and the land [25]. Harris [23] notes conflict between authors surrounding the importance of traditional therapeutic boundaries, as Totton [26] claims boundaries might interfere with the therapeutic relationship, compared with McKinney [9], who holds value in these structures. McMullan [27] considers nature’s rules demand the therapist and client to play by a new rulebook, which cannot always be controlled. For McKinney [9], nature’s control does not affect the rules transferred from traditional therapy, although it presents an ability to be casual and offer a less intimidating form of therapy. The therapist can adapt therapeutic activities in new contexts and re-configure their role, skills, and abilities. Conversely, some write of the mutual benefit of restoration and nurture in outdoor practices, allowing the therapist to remain separated from client material and avoid burnout [1].

This research investigates the therapeutic use of ‘the outdoors’ within outdoor therapy by exploring the process and embodiment in relation to other psychotherapeutic practices. Further, the study seeks to extend IPR methodology to gain an in-depth account of the lived-experience of UK-based outdoor therapy practitioners. 

## 2. Methods

### 2.1. Interpersonal Process Recall

A process-focused interview technique, interpersonal process recall (IPR), was used to collect data. This unique methodology was applied to gain an in-depth and contextualized account of the practitioner’s reflections of an outdoor therapy session. IPR allows the inquirer (researcher) and recaller (participant) to come to a common understanding about the recaller’s experience [28]. Created for corporate use, Kagan [29] developed IPR to understand professional responses for training purposes. Inskipp [30] later introduced IPR for counselling training, which has led to its application in reflective practice and studying therapeutic interactions [31]. Although IPR has been reported as an effective means of investigating therapeutic processes, there is limited research applying its methodology [32]. Brown et al. [33] advocate IPR as a person-centered design that increases the participant’s reflection and involvement. Kettley et al.’s [28] account of IPR offers a rationale for its philosophical congruence with phenomenological and person-centered approaches, with a particular emphasis on enabling participant-led research. 

IPR captures a qualitative-rich, in-the-moment, and specific account of interactions and processes [34]. McLeod [35] (p. ix) relates qualitative research to psychotherapy, which gains “holistic, nuanced, personal, contextualised, incomplete” data. IPR assumes that within the moment, multiple thoughts, feelings, and sensations are experienced but not necessarily recognized [36]. Whilst Macaskie et al. [37] note IPR recalls individual’s conscious but often unprocessed thoughts, Finlay [38] (p. 10) recognizes “sometimes it languages things we already know tacitly but have not articulated in depth. At other times, quite surprising insights reveal themselves.” A recent study found IPR allowed the researcher and participant to actively share the meaning-making process and co-construct research conversations [37]. The process-focused interview extracts insights through observation and direct questioning as the recollections unfold, paying close attention to context [34]. Kettley et al. [28] note the parallels between phenomenological studies which seek transparency and person-centered practices, which advocate congruence. IPR could offer new ground to explore a practitioner account that is embodied, nuanced, and contextualized and a method which engages the participant to become actively curious and reflective of their personal practice.

### 2.2. Sample

Phenomenological studies use small homogenous samples to examine convergence and divergence [39]. Purposive sampling allowed for three participants to be selected for their suitability [40]. All participants were registered or accredited counsellors of a Professional Standards Authority with between 7 and 20 years of experience as practitioners:

Participant A: Works with individuals indoors and outdoors with a person-centered modality and uses various outdoor sites from local parks to mountainous regions (male).
Participant B: Works with individuals indoors and outdoors following a person-centered modality within a pluralistic agency, using woodlands, parks, and fells (male).
Participant C: Works with groups and individuals in indoor and outdoor venues, using an integrative approach combining Gestalt, Jungian, transactional analysis, and person-centered theory and aspects of coaching and wilderness therapy (male).

### 2.3. Informed Consent

Participants received information regarding the process, aims, contribution to research, and right to withdraw [41]. Within a very small industry of outdoor therapists within the UK, participants were warned that despite the appropriate use of pseudonyms and the removal of sensitive and place-specific data, they may be recognizable through their narrative.

Bond [42] warns researchers in counselling and psychotherapy that client confidentiality could be compromised with in-depth data. As a result, the research participants were briefed to maintain client anonymity and given the opportunity to read and censor sensitive data from the transcripts as encouraged by Henry and Fetters [43]. “Honouring any promises about confidentiality carries special ethical weight because this is central to practitioner and researcher trustworthiness in this field of work” [42] (p. 7).

### 2.4. Procedure

To respect client confidentiality and avoid interference to the client’s therapy, this research focused on the therapist. Data collection involved a three-step procedure:

Step 1: A face-to-face initial-interview: To gain background and contextual data on the participant’s practice and philosophy of outdoor therapy. Digitally recorded and transcribed (45 to 60 min).

Step 2: Post-session-reflective-recording: Using a semi-structured list of reflective questions, participants remotely recorded their immediate reflections after an outdoor therapy session to gain an uninhibited account of the participant’s lived-experience. Recorded (participant’s smart-phones) and transcribed (30-minutes).

Step 3: Face-face IPR-Interview: The participant and researcher listened to the post-session-reflective-recording together at the participant’s working location. The researcher and participant were able to pause the recording at points of interest to gain depth, perspective, and insight (60 to 90 min).

In IPR, “Interviewees are cued to remember various reactions and ideas that occurred during the session but might not readily come to mind unassisted” [34] (p. 1). “The IPR process slows down the interview conversation, giving interviewees time to meditate on and verbalize complex experiences” [34] (p. 3). Similar to Brown et al.’s [33] study, the post-session-reflective-recording was used as a stimulus for reflection, to replay and invite participants to pause and recall thoughts and feelings not commented upon within the original recording. This allowed for a deeper understanding of the subjective experience, a point of reference to gain perspective upon, and gave voice to participants to re-encounter their account. This multi-layered approach offers a unique methodology of examining the phenomenon.

### 2.5. Ethical Considerations

Ethical approval was granted by the University of Worcester [44] and reflects the British Association for Counselling and Psychotherapy’s research ethical guidelines [45] and the Economic and Social Research Council’s [46] ethical guidance framework.

### 2.6. Researcher Bias

Whilst psychology is concerned with the unavoidable presence and meaning systems inherent to the researcher, IPA embraces the relationship between researcher and subject matter [39]. The researcher’s personal bias stems from involvement as an outdoor educator, integrative counsellor, and individual using the outdoors restoratively.

### 2.7. Analysis

Interpretative phenomenological analysis (IPA) offers a qualitative approach to investigate participants’ experiences, examining how people make sense and understand the experience in its own terms [47]. As such, IPA is often used alongside interviews to “recall the parts and their connections and discover this common meaning” [39]. This experiential approach invites the researcher to engage creatively with the participants’ reflections [39]. Considering the interdisciplinary theories related to outdoor therapy, the ideographic nature of IPA is called upon to understand “what the experience for this person is like, what sense this particular person is making of what is happening to them” [39] (p. 3). This appears appropriate for outdoor therapy as “services in this field (are) using different language to describe their activity and benefits, operating different delivery models and using different measurements of impact” [5] (p. vi). Whilst this research favors a qualitative in-depth methodology to explore outdoor therapy practice, Smith et al. [39] warn exploratory and interpretative research findings should not be regarded as exhaustive but can generate new areas for inquiry. IPA involves iterative analysis, moving back and forth at different ways of looking at data, rather than sequentially [48]. A major principle of phenomenology is to move past taken for granted assumptions and discover the essence of experience [49,50]. Like many strands of humanistic counselling, phenomenology regards participants as the expert of their experience and warns researchers not to re-word or label extracts [49]. Allen-Collinson [49] advises researchers to include original extracts to speak for themselves, record researcher conceptualizations, and use triangulation to validate findings. This research follows Smith et al.’s [39] (p. 84) guidelines, dissecting the transcript using:

“Descriptive comments focused on describing the content of what the participant has said, the subject of the talk within the transcript… Linguistic comments focused upon exploring the specific use of language by the participant… Conceptual comments focused on engaging at a more interrogative and conceptual level”.

From these descriptive, linguistic, and conceptual comments, emergent themes were generated before examining the cases and searching for connections across themes and abstracting patterns across cases to form super-ordinate themes [39,51]. Owing to the multi-layered analysis, the research was manually coded as the researcher’s preferred coding method. The researcher had previous experience of IPA as a post-graduate student. This process involved reading, re-reading, familiarization, immersion, and incubation through continued engagement with the recordings and transcripts [52]. The researcher made notes of codes and then themes through abstraction; putting like with like, subsumption; identifying a theme which acts as a magnet to other themes, and polarisation; focusing on the difference between themes rather than similarities [39]. Whilst this process provided opportunity to engage with data in different forms, Smith et al. [39] recognize the researcher often moves into deeper stages of interpretation, whereby they begin to understand the data. The super-ordinate themes arrived at offer a compromise between a systematic and intuitive analysis process, which reflects not only the participants’ lived-experience but also the researcher’s interpretation [39,53].

## 3. Results

The original research submitted to the University of Worcester included five themes. This paper presents one theme: ‘Transitional landscapes—transitional thinking’. This theme was chosen as it best demonstrates the application of interpersonal process recall in gaining an in-depth and nuanced understanding of the environment within the session. The theme is broken into subthemes including; the embodied experience, parallel processing, and watching for drift.

### 3.1. Transitional Landscapes; Transitional Thinking

Outdoor therapy can be direct (working outdoors from the start), planned (starting indoors with a plan to move outside), combined (using indoor and outdoor spaces on alternate or particular sessions), or emergent (finding opportunity for the work to progress to an outside space). Emergent opportunities arise where the client learns and becomes interested in an outdoor approach or where the practitioner gets a sense that working outside might be safe and beneficial to the client. As with traditional counselling, the initial sessions are important for establishing the therapeutic relationship, ensuring there are clear and contracted boundaries of practice and establishing the focus of the work and whether the practitioner’s modality will suit the client. 

Compared to indoor counselling that is often assisted with a clear transition from the waiting room to the contained counselling room, in a direct approach, where the client and practitioner meet and start working outside from the beginning, there is a less clearly defined transition. Participant C describes one approach using the environment and assisting the client to make a transition between landscapes and beginning the therapy session.

“We’d get to the bridge at the head of the lake … that’s like a passage and I’d say to people… when we come off the tarmac road I’d invite them to think about their leaving one kind of environment and going into somewhere else”.

(Participant C)

This approach uses a land feature to symbolize the transition into the therapeutic session. It indicates that for participant C, the session offers an escape from everyday life and a passage into an alternative space. The journey becomes metaphoric as well as physical as the participant transitions from one space to another. Conversely, participant B describes a combined approach whereby the outdoors is used as an experiential space to explore the therapeutic work.

“He chose a route through some paths, woodland paths and ended up going off track and over walls… it was almost quite playful, and quite a sense of lostness and re-emerging and all that kind of stuff he was experiencing which mirrored some of our indoor sessions, literally as opposed to metaphorically”.

(Participant B)

In this example, the client is able to actively experience some of the metaphoric content of an indoor session, the metaphors of being lost and finding themselves are given a literal meaning as the client navigates through the forest. A combined approach allows for the client and practitioner to work with the presenting issue through rational, reflective and abstract forms.

As the sessions progress, the client may become more confident to work with the practitioner and the outdoor approach. The sessions transition from beginnings (getting comfortable with the approach) to middles (utilizing the approach to explore the presenting issue). Participant B describes a client’s integration of the natural environment within the session. The client starts to experience an embodied agency within the outdoor environment.

“He was moving along, like in the same way that his emotions were moving… feeling very lost, very confused… what mirrored that process was walking along in the light, a light airy space for a little bit and then going through the woods as per his direction, and getting very lost and weaving our way through these little paths”.

(Participant B)

Here, the therapeutic process emerges with, and is guided by the natural environment; as the client and practitioner talk, the client is able to move into spaces of shade or light, clear pathways, or trickier terrain. The terrain affects the conversation as the natural environment stimulates the therapeutic process, providing dynamic material within the session. Equally, the client can affect the terrain by changing the path they choose; thus, enabling an embodied expression to emerge. The practitioner observes the client shifting between affecting and being affected by the environment. The practitioner’s role shifts, allowing room for the natural world to interact within the therapeutic relationship.

“Just at the point where we were more tangled was when we could actually start to see the sky through the trees again…and then saw the hope, the light through the trees and that seemed to help facilitate him getting back to himself, answering his question about the here-and-now”.

(Participant B)

Whilst the client can dictate the path, they are also in a dynamic and emergent terrain. After leading the way into a thick mass of trees, the environment offers a natural window and sense of perspective. The light through the trees offered a symbol of hope and provided light to the situation that shifts the client’s thinking process. This is experienced physically, emotionally, and cognitively as the client finds patches of clarity within an enclosed forest. The client is able to discern the figure from the ground and return to the present moment.

“That for me is like the holy grail, when the experience of the session and the experiencing of it feels as real as what’s going on internally, we hit those moments throughout that journey because the client is picking the route in tune with the content of their session”.

(Participant B)

The practitioner’s likening of an embodied session (synchronicity between mind and body) to the holy grail indicates a sense of actualization, flow, or epiphany that is deep and powerful. To the practitioner, the client’s ability to work in this way and encounter such a state of mind indicated that the session was meaningful. The practitioner’s role is to dynamically facilitate this engagement with the natural environment and work with the client to offer awareness.

Being with the client outside allows other-than-spoken processes to emerge. Participant A explains that silently walking with the client was equally as useful. The session takes a different pace and allows the process of walking to hold the space between conversations.

“I think walking gave us an opportunity to share times of stillness and silence which were sometimes necessary for my client to be able to process what was going on and to find the words to say what he wanted to say”.

(Participant A)

Transitions in outdoor therapy take many forms. These transitions include the intentional shift from an indoor to outdoor space, the client’s attunement and integration of the approach, and the shift of the practitioner to provide space for the natural environment to interact and be an active component of the work.

### 3.2. The Embodied Process

Outdoor therapy reframes the therapeutic relationship and offers both the client and therapist a different experience of one another. This reframe symbolically alters the perception of the role and context of the professional. The therapeutic work becomes defined, negotiated, and maintained within the context of the outdoor environment. The therapeutic process takes on additional dimensions as the client and therapist move through and engage with the environment. Participants noticed that working outdoors impacted their experience of the client:

“You feel kind of more what they’re feeling and their kind of anger can become perhaps more understandable or certainly experienced anyway!”.

(Participant A)

Outdoor therapy offers a holistic approach. Whilst indoor counselling works mainly with the cognitive and emotional, outdoor therapy involves an active element that invites clients to be present with their emotions, thoughts, and actions. The immersive experience can impact the practitioner’s ability to experience the client authentically.

“In a therapy room… they can see the clock… but in nature when they’ve been walking around in the woods and they don’t really know where they are, old worries and anxieties and things may well resurface but they may be reflecting the real person rather than the person they try to be”.

(Participant C)

Participant C explains that outdoor therapy allows clients to become immersed in the moment, and in doing so, they might forget about how they are trying to portray themselves and start being authentic. Participant C suggests this process may lead to worries and anxieties resurfacing, which offer a more genuine experience of the client. Working with the client in an experiential way enables the therapist to observe and experience the client’s way of being in real situations rather than through the client’s self-reflection. This allows the therapist to engage with the client’s authentic self and provides an opportunity to experiment with coping-mechanisms.

“If they don’t look after themselves physically in that environment, then what does that say about them emotionally? Are they able to take care themselves?”.

(Participant C)

Outdoor therapy alters what it is to engage in therapy and for participant A, reframes the purpose of therapeutic encounters from clinical to organic.

“Stillness’s and silences can seem a very natural part of the process of walking, whereas in a counselling room, sometimes those dark silences can seem very, yeah unbearable almost”.

(Participant A)

In this example, the participant reflects upon the meaning implied by stillness and silence. He suggests the tone of silence is altered when walking to resemble a natural pause, whereas within a counselling room, the tone can feel imposing and stifled. Equally, participant A reports an ability to experience their client’s disconnect, their discomfort and vulnerability, and the impact of this on their work.

“One particular client… it was very obvious there was not psychological contact between him and his surroundings… within about ten-fifteen minutes I had this most enormous headache… it was really frustrating because I was really feeling that sense of complete disconnection with where I was… I was in his world, I’d kind of lost a sense of me as a counsellor… I was as disembodied as he was”.

(Participant A)

Participant A details a disconnect between the client and their surroundings, which in turn affects the practitioner’s ability to connect with their environment. This disembodiment affects the practitioner’s sense of self. Whilst the practitioner uses the natural environment to remain grounded and focused on the client, here, the practitioner is unable to make psychological contact between nature–practitioner–client.

When removed from the traditional context of counselling and engaging experientially in outdoor therapy, the practitioner must be cautious to remain focused, rational, and professional and avoid getting lost in the experience:

“The risk is you have a genuine relationship with somebody… then you actually feel their pain and their sorrow and their sadness”.

(Participant A)

Participant A considers the risk of intimacy on professionalism. He suggests practitioners working outdoors might have an altered perception of the role of intimacy in the therapeutic relationship and be more inclined to experience their clients authentically. This suggests that for participant A, the risk of intimacy is not that professional boundaries will be compromised, but that the practitioner may begin to feel their client’s emotions.

“I think that’s one of the reasons why counsellors are very reluctant to work outdoors because… strangely… it seems paradoxical because what you want is intimacy, I think often counsellors are actually very scared of true intimacy”.

(Participant A)

Participant A identifies a paradox whereby on the one hand the work between the client and practitioner fosters intimacy within the working relationship and on the other, professional boundaries imply that true intimacy is to be un-boundried or step over the professional boundary of practice. For participant A, professional boundaries do not restrict intimacy, nor does intimacy restrict professionalism. There is an indication that counsellors may be restricting their work through limiting the intimacy within the working relationship and that by situating work too squarely within professional boundaries, the innate human connection is lost. Despite this, participant A acknowledges that intimacy must be managed with care.

“I think trusting relationships can develop very quickly, that can also be a problem too in the sense that sometimes people might be working quicker than they actually feel comfortable with”.

(Participant A)

Here, the participant explains that intimacy takes time to develop between the client and practitioner. The pace, intimacy, and depth of the work are managed in the altered context. The practitioner must consider the duty of care to the client and decide what is appropriate and best for the client within the scope of the approach.

### 3.3. Parallel Processing

The outdoor environment provides a dynamic element affecting both the client and practitioner. The participants expressed a motivation and passion for outdoor environments as a place of self-care. These places become a working environment offering a symbiotic relationship and providing restorative conditions for both the therapist and client and a sense of rejuvenation to the therapeutic work:

“I notice that when I’m outside I can be more immediate with what is going on in the moment, I can be more focused, perhaps more available for the client… that has an impact in terms of holding from a person-centered point of view… holding of the necessary and sufficient conditions”.

(Participant A)

Participant A describes a sense of attunement to the client within the natural environment. The participant describes a sense of seeing more within the moment and being grounded in the present here-and-now in which the client is the center of attention. Here, the person-centered core conditions (empathy, congruence, and unconditional positive regard) flow naturally between nature–practitioner and practitioner–client to provide the conditions for therapeutic change. Not only is the client held, nurtured, and contained, but the practitioner too. However, the therapist must be aware of their own processes and motives within the session, putting aside their ‘stuff’ to be present with the client.

“My feeling is joy, I’m finally in a new place, there’s a new lostness; I love exploring so for me there’s an adventurous side, I love that feeling. But I love it so much that I’ve had to learn how to not let that get in the way of how the client is feeling… this has taken a long time… to both feel that excitement that I’m having in the moment… but to be with the client and how they’re experiencing that moment”.

(Participant B)

Participant B describes his emotional response to the sense of lostness within the session. He acknowledges his inner-reaction and sense of adventure which is parked to remain present and attuned to the client’s experience. Participant B indicates a journey of realization and training that he has taken to remain present with the client and to sustain focus during the session.

Equally, the process of joining with the client and remaining responsive to the terrain and safety elements requires the therapist to dynamically examine their anxieties and intrinsic response to the land in relation to the context of the work and their code of practice;

“I keep feeling naughty about that… like little school boys playing… we were in this deep process literally a moment ago, but it got really steep and really windy, I had this feeling like ‘we shouldn’t be here’… and I just have to let it go because I’m looking at the client just carrying on talking but he’s weaving through”.

(Participant B)

Participant B’s use of the words ‘naughty’ and ‘school boys’ indicates a more playful dynamic between the client and practitioner. The participant uses ‘we’, suggesting that the moment was a shared experience and state of being. The practitioner notes the change in the terrain and its impact on their movement. Here, the practitioner takes a moment to check-in and acknowledge his sense of discomfort with the situation before considering its impact on the client’s safety and process. The practitioner is able to focus on the client and reserves his doubts to allow the client’s process to continue. As a person-centered counsellor attuned to following the client, participant B explains the practitioner must recognize and hold their own agentic response to nature. Dissonance can emerge between the client’s and the practitioner’s experience.

“For me it was divine, it was heavenly, but for my client it who was feeling very suicidal at the time, he just had this deep feeling of foreboding because it was just too much”.

(Participant A)

Participant B offers another example, whereby their passion and motivation for the outdoors was not reflected by the client. In this case, the practitioner was forced to consider the intention behind the approach and who was benefiting from the approach.

“I was expecting them to have the same relationship to nature as I did. Which was enthusiastic, love, joy, it was amazing the best thing in the world and the first person I took outside hated it… I was really disappointed”.

(Participant B)

Participant B reflects upon how he has attempted to narrow the gap between his personal experience and the client’s experience using a process of intentional disorientation, within a safe and confined boundary, to become more equal, avoid complacency, and better understand the here-and-now experience.

“I didn’t realize until I did this on reflecting on this… I’m aiming for this ideal kind of equality with the client and the session… to mirror what I’m actually doing indoors… I wanted to actually go somewhere I hadn’t been before, so that it did feel more like it does in a normal session which is new territory, new ground”.

(Participant B)

Taking therapy outside requires the practitioner to be comfortable and aware of their own relationship with outdoor and natural spaces. Their competence and comfort in these environments allow them to be present with their client’s experience. Staying in tune with their own response, the practitioners internally supervise the session, considering the client’s wellbeing and the therapeutic work aside the landscape and terrain.

### 3.4. Watching for Drift

Working with the client’s response to nature requires and invites the therapist to experience additional roles and blurs the boundaries of the traditional therapeutic hour. This offers multiple elements for the practitioner to balance and manage simultaneously. Participant A describes the importance of finding safe conditions for the session to emerge. Where the conditions are not suitable, the practitioner adapts accordingly until conditions are met.

“Walking to the park, we would have general sort of chit-chat but we wouldn’t be doing sort of deep work because I’d end up walking into a car”.

(Participant A)

Once safe conditions are found, the practitioner can settle into the session. Whilst the practitioner continues to dynamically assess safety, participant B describes an experience of becoming immersed in the session with the client, presenting a risk of drift from the presenting issue to the experience itself.

“I’m almost giggling here actually because I remember… there was a part where the alliance was as if we were being a bit naughty like here we are doing a counselling session, talking about all these things and then we find ourselves weaving up, weaving up quite a steep track, not even a track, a steep wall with no track”.

(Participant B)

This can alter the therapeutic alliance, whereby the client and practitioner experience each other differently.

“That pretense goes, you just lose yourself… we shared in those moments so that our eye contact was more and we were having fun”.

(Participant B)

The therapist shifts focus with the terrain of the session. Whilst managing safety, the therapeutic work, the client’s experience, and the environment, the outdoor therapist must also follow their navigational location. Participant B describes a moment where he did not know their exact location and considers the impact of this on the client.

“I didn’t know that was the way out, he did actually find it… he thought I was pretending… that I did really know where I was… and I didn’t. And that was really levelling”.

(Participant B)

This can alter, shake or destabilize the client’s view of the therapist and the perceived competence, safety, and professionalism bestowed upon the practitioner.

Watching for drift requires the practitioner to juggle the different hats that they must wear to work as competent lone-practitioners. Whilst working in line with their ethical framework and seeking supervision to review their work, practitioners must be mindful of the heightened duty of care they have for clients whilst outdoors.

## 4. Discussion

This study adopted an interpretative phenomenological analysis to explore the participants’ lived-experiences of outdoor therapy sessions. The theme presented reflects upon the use of an environment which is intrinsically therapeutic and which can lead to transitional thinking [54], and the multiphasic nature in which cognitive and psychological states ebb and flow throughout the encounter [55]. This research supports links between internal and external landscapes [56], symbolism between nature and the therapeutic alliance [12], the other-than-human-world and the reflective process within the session [25,26], and the impact of sharing external landscapes upon the therapeutic relationship [26]. There is also support that the mechanism of change depends on the therapeutic modality of the therapist [23].

This research builds upon Revell and McLeod’s [1] account that the altered physicality and embodied relating between the client–practitioner/client–nature/mind–body can create opportunities for synchronicity, metaphors, and transitional experiences to emerge. There becomes a balance, whereby the practitioner must step back to let the client lead whilst containing the safety, focus, and depth of the session. The practitioner holds the process, noticing the client’s physical and verbal expression, transition between states, and interaction with surroundings. Further, the practitioner notes whether the client is affecting or affected by the environment and helps to explore the links between the internal and external, delicately managing the figure and ground. The figure and ground present “ambivalent and nuanced spaces [with] many shades of meanings… perceived as healthy and unhealthy at the same time” [57] (p. 261). The natural world provides texture, context, and stimuli to explore the figure and ground through physical, cognitive, and emotional modes.

In a process of multi-sensory involvement, the therapist becomes part of the experience, moving between witness and companion within the client’s process. The therapist watches for drift from the presenting issue, aware of experiences which might become un-boundried or destabilize the process. As Baer and Gesler [58] advocate, the therapeutic potential of environments changes over time and therapists must assess the validity of the landscape on the healing process. This was confirmed as participants explained the selection, evolution, and therapeutic use of sites.

This theme builds upon previous literature, which identified the positive effect on therapists’ personal psyche and ability to prevent burnout [1]. The altered therapeutic relationship is examined in relation to the impact of experiencing the client; Revell and McLeod [1] identify a process of bodily empathy, whereby therapists experience their clients more holistically. Whilst Revell and McLeod [1] note a freer and less inhibited relationship that emerges, altering the dynamic as the client and therapist move from face-to-face to side-by-side, the findings suggest the therapist is not completely uninhibited and care-free and works alongside a complex process of providing an appropriate therapeutic relationship, maintaining flexible boundaries, and being able to separate and hold their own ‘stuff’ apart from the client.

Like Jordan and Marshal [24], the study found the neutral space allows the therapist to be more real within a natural setting and provides deepened intimacy, although they caution that intimacy must be handled carefully. This offers an opportunity to experience the client in real time and witness the client’s disconnect, discomfort, and vulnerability. This can widen the gap between the client and therapist’s experience, allowing the therapist to work with the client through the issue or alter the therapeutic experience as necessary. Jordan and Marshall [24] note the ability for the experience to provide immediacy for both the client and therapist. The findings support an altered therapeutic alliance and therapeutic role in terms of bringing more of themselves into the relationship and loosening their professional role [1]. Berger and McLeod [12] (pp. 87–88) identify the role of the therapist as “witness, container, and mediator” shifting in relation to the client’s engagement with nature. In this case, it appears the therapist can also become a ‘partner’ with the client, experiencing together. In many cases, participants detail processes which are adapted from indoor counselling. This appears to align with McMullan’s [27] considerations that the alliance is removed from traditional rules of therapy, instead locating and obliging nature’s rules. However, as Harris [23] warns, participants equally detail the ability for the alliance to become destabilized based on the client’s expectations of the therapist not being met or due to lacking boundaries.

### 4.1. Limitations and Reflexivity

The sample inclusion and exclusion parameters had specific demands of the research participants. Whilst these were upheld, an unexpected element was the scope of participants’ work and range of sessions, which presented within the post-session-reflective-recordings. Such diversity is echoed in Harris’s [23] research, which underestimated the range and complexity of cases presented. The diversity of cases proved difficult to hold amongst one another. For example, holding group work amongst one-to-one therapy or overnight sessions amongst 50-minute sessions. An implication for future IPR research is to specify both participant and post-session-reflective-recording parameters.

In accordance with a phenomenological approach, this research explored the thing itself, applying IPR research methodology providing a reflective stance for the participant and researcher and generating practitioner knowledge. Each stage in the IPR procedure allowed a different layer of depth to be explored and highlighted different aspects of the lived-experience. Whilst it might be argued that the findings lack generalizability, this research questions the extent to which generalized findings would benefit the field of outdoor therapy and considers it critical to know more about specific practices.

### 4.2. Implications

An alternative view of the therapeutic alliance was encountered whereby the practitioner and client become partners and can reveal their authentic selves. The therapist is both a participant in the experience and holds responsibility for the therapeutic encounter. The relational dynamic appears complex and needs to be considered from the client’s perspective.

In addition to the many positive accounts of outdoor therapy, investigation needs to explore the experiences which drift from the therapeutic aim, distract from the goal, or destabilize the therapeutic process and the implications of such occurrences. In view of physical and emotional risk, and the reporting culture of the counselling and outdoor industries, further research might investigate the provision of support extended to lone-practitioners.

## 5. Conclusions

This research offers insight into outdoor therapists’ lived-experience and practitioner knowledge within a specific outdoor therapy session. At the outset, the research intended to understand both the embodied experience and therapeutic process. What emerged was a detailed account of the synchronicity between the two as the therapist receives and seeks input from the natural surroundings. The findings progress from the therapist’s philosophical stance, motivation, and theoretical position to a contextualized and practical understanding of the process. The data reveals the therapist’s choice of therapeutic sites, impact of physicality on the dialogue, and use of the outdoor context. The IPR-interview distinguishes the therapist’s perceptions for their clients and their own lived-experience and how these states are altered in transitional landscapes. These findings highlight the significance of an altered therapeutic partnership and the impact of parallel experiencing upon the therapeutic encounter. These factors were considered in relation to the therapeutic frame and the practicalities and difference of working outdoors.

Whilst acknowledging limitations presented by a diverse sample, IPR offers a tool for future research, enabling both the participant and researcher an experiential and reflective stance. Further research is needed on the client’s lived-experience, and an understanding of the process and embodied-experience. An understanding of positive and negative experiences could inform practice, and offer insights for appropriate training and supervision, and generate constructive dialogue amongst outdoor therapists.
